# Commentary: Executive functioning—a key construct for understanding developmental psychopathology or a ‘catch-all’ term in need of some rethinking?

**DOI:** 10.3389/fnins.2017.00130

**Published:** 2017-03-17

**Authors:** Timothy R. Rice

**Affiliations:** Department of Psychiatry, Division of Child and Adolescent Psychiatry, Icahn School of Medicine at Mount SinaiNew York, NY, USA

**Keywords:** executive function, emotion regulation, child development, psychoanalysis, psychodynamic psychotherapy

## Introduction

In a recent editorial (Halperin, 2016) Jeffrey Halperin calls for a firmer grasp of the executive function construct and its boundaries. His goal is to advance knowledge of the neurocognitive underpinnings of developmental psychopathology. In this commentary I propose that the development of the emotion regulation construct in relation to executive functioning is an ideal pursuit of this aim.

Prior writings (Rice and Hoffman, 2014; Rice, 2016a,b,c) develop the conceptualization of implicit emotion regulation through analogies with defense mechanisms. Observed similarities between “hot” executive functions, implicit emotion regulation, and a contemporary understanding of defense mechanisms (Rice, 2016b) facilitates a more textured understanding of executive functions. Articulation and analysis of the differences between these three conceptualizations of different fields and frameworks performed in tandem with reflection upon their similarities yields an opportunity to develop our understanding of all three concepts in the pursuit of a more nuanced understanding of developmental psychopathology.

## Emotion regulation and executive function deficits

The role of emotion regulation deficits have become more apparent in a wide range of childhood psychopathology, most notably attention deficit hyperactivity disorder (Graziano and Garcia, 2016), and oppositional defiant disorder (Cavanagh et al., 2017). The creation of disruptive mood dysregulation disorder (Baweja et al., 2016) and ongoing controversies regarding childhood bipolar disorder (Towbin et al., 2013) both suggest a benefit to a more developed understanding of emotion regulation processes in children.

The broad construct of emotion regulation (Gross, 2013) has been significantly developed through the introduction of a differentiation between explicit and implicit emotion regulation deficits (Gyurak et al., 2011). Explicit emotion regulation refers to those that demand conscious, effortful application, while implicit refers to those that proceed automatically and unconsciously. These two branches have been shown to have distinct neural correlates (Etkin et al., 2015), with implicit emotion regulation exhibiting a greater reliance upon ventromedial regions of the prefrontal cortex. These regions include the orbitofrontal cortex (OFC), ventromedial PFC (vmPFC), and ventral anterior cingulate cortex (vACC). Explicit emotion regulation is more reliant upon dorsolateral areas including the dorsal anterior cingulate cortex (dACC) and the dorsolateral PFC (dPFC; Etkin et al., 2015).

The identification of implicit emotion regulation as a differentiated branch enables comparison with the “hot” executive function construct. “Hot” denotes automaticity and rapidity and neuroanatomically includes ventral prefrontally-mediated automatic and effortless modulation of limbic and visceromotor areas (Zelazo and Carlson, 2012). In addition to conceptual similarity, these neural correlates show high similarity with those of implicit emotion regulation (Etkin et al., 2015). Reflection upon the similarities and differences permits a more nuanced understanding of both.

## Defense analysis

Similarly, reflection upon the psychoanalytic construct of defense mechanisms in relation to these two constructs is fruitful. Contemporary child psychoanalytic psychotherapy involves the interpretation of children's observable defenses against unwelcome affects (Bornstein, 1945, 1949; Becker, 1974; Hoffman, 2007). In place of reliance upon the metapsychology of drive theory, contemporary clinicians understand defense mechanisms as unconscious, automatically-implemented processes to regulate negative emotions. There is thus conceptual similarity between implicit emotion regulation and defense mechanisms (Rice and Hoffman, 2014). This commentary clarifies for the first time in the literature the added similarity between defense mechanisms and the executive functions through the “hot” executive function construct.

This neurophysiologically-baesd comparison yields a contemporary, brain-based foundation to earlier conceptualization of the ego and executive functions (Dyrud, 1969). There is benefit to reflection upon the specific ego function of defense mechanisms and the “hot” subset of executive functions. This advances Dr. Halperin's goal while not failing to appreciate the differences between these constructions and submitting to reductionism.

For instance, imagine a clinical scenario in which a school-aged child in foster care is playing catch with a clinician. When the clinician comments that the session is soon to end the child begins wildly throwing the ball into the tiled dropped ceiling with force, creating loud banging noises. The child's violence places the clinician at unease.

The clinician's commenting to the child that the dysregulated play was preceded by the comment that the session was soon to end helps the child to see the self-protective purpose of the dysregulated play: the child places the unease onto the clinician and so turns a passive stance into the active. Also conveyed is the failure of these underdeveloped implicit emotion regulation and “hot” executive function strategies to regulate disavowed and painful affects. The feelings of grief, loss, and longing that are so sensitive to the foster child are recalled by the impending end to the session. The child shows the maxim that it is easier to go on the offense than to be on the defense. Instead the clinician feels the unsettledness while the child enjoys the destructiveness of aggression.

After commentating on the link between the event and the behavior and thereby creating a causal connection through unconscious processes, the clinician may comment further on any of these mechanisms through simple, experience-near language, like, “It's easier to make loud banging noises than to feel powerless to your wild and crashing feelings.” Through iterative intervention the child learns to reflect upon patterns and to create a space to develop alternate means of emotion regulation, including recognition and verbalization of appropriate affects and to engage in a direct confrontation with their salience and personal importance.

## Conclusion

Making the similarities between implicit emotion regulation, “hot” executive functions, and defense mechanisms while preserving respect for their differences offers a unique opportunity. Dr. Halperin's call to explore the character and boundaries of the executive function construct is followed when subsets of executive functions are considered in relation to alternative models of neuroscience as well as common clinical care. The realization that unity exists alongside differences creates a more nuanced understanding of executive functions as well as of the affective neurosciences and psychoanalytically-informed clinical care.

## Author contributions

The author confirms being the sole contributor of this work and approved it for publication.

### Conflict of interest statement

The author declares that the research was conducted in the absence of any commercial or financial relationships that could be construed as a potential conflict of interest.
